# Factors Associated With Individual Emergency Preparedness Behaviors: A Cross-Sectional Survey Among the Public in Three Chinese Provinces

**DOI:** 10.3389/fpubh.2021.644421

**Published:** 2021-05-21

**Authors:** Ning Ning, Man Hu, Jin Qiao, Chaojie Liu, Xiaowen Zhao, Wei Xu, Weilan Xu, Bin Zheng, Zhiqiang Chen, Yi Yu, Yanhua Hao, Qunhong Wu

**Affiliations:** ^1^School of Health Management, Harbin Medical University, Harbin, China; ^2^Collaborative Innovation Center of Social Risks Governance in Health, Shanghai, China; ^3^Cancer Hospital of the University of Chinese Academy of Sciences, Hangzhou, China; ^4^School of Psychology and Public Health, LaTrobe University, Melbourne, VIC, Australia

**Keywords:** individual emergency preparedness behaviors, emergency management, structural equation model, cross-sectional survey, risk perception

## Abstract

**Introduction:** This study aimed to identify factors associated with individual emergency preparedness behaviors which play an important role in effective emergency response.

**Methods:** Data were drawn from a cross-sectional survey conducted in China's Heilongjiang, Guangdong and Sichuan provinces in 2017. Questionnaires were administered through face-to-face interviews, and 2,506 were valid for data analyses. A structural equation model was established to test the direct and indirect effects of the relevant factors on individual emergency preparedness behaviors.

**Results:** Low levels of emergency preparedness were found: 28% of respondents reported being fully/partly prepared. The attitudes of the respondents toward emergency preparedness had the strongest association with emergency preparedness behaviors, with a total effect of 0.483. This was followed by self-efficacy (0.305) and training/exercise (0.295). Risk perception had the weakest effect (0.045) on emergency preparedness behaviors.

**Discussion:** Improving attitudes of the public as well as their ability to prepare for emergency events is important for effective emergency management.

## Introduction

Over the past decade, the world has witnessed a dramatic increase of life and economic loss arising from emergency events, such as Hurricane Katrina, the Wenchuan earthquake in China, and the Ebola outbreak in West Africa (1). These emergency events have also led to serious physical and mental health consequences for those who survived.

Although emergency events usually occur suddenly, appropriate preparedness can mitigate the hazards caused by emergency (2). Emergency preparedness requires actions from both institutions and individuals. Individual preparedness behaviors play an important role in an effective response (3, 4). During the emergency, individual households have to take action before any organized response kicks in, in order to minimize loss and damage (5).

Previous studies have found that individual emergency preparedness was lower in China in comparison with some other countries (6, 7). In Japan, first aid kits are widely available in living, study and work settings (8). The USA established a specialized agency FEMA (Federal Emergency Management Agency), providing knowledge and funding support to state and local governments for their efforts in emergency preparedness. It was reported that 44% of families in the USA have developed a household emergency plan (9).

Empirical evidence shows that individual emergency preparedness behaviors change with knowledge, attitudes, risk perception, and self-efficacy (10, 11). However, it is not clear about how these factors are influenced by external forces. Several studies claimed a positive association between past disaster experiences and better emergency preparedness; but such a claim was rejected in a few other studies (12). Most emergency management plans contain a component of drill exercise. It is generally accepted that drill exercise can improve emergency preparedness (13). Overall, there is a paucity of literature documenting the mechanism of individual emergency preparedness behaviors.

This study aimed to identify the factors associated with individual emergency preparedness behaviors. We chose structural equation modeling as the method by which to explore the potentially expansive web of interrelationships between the various factors.

There are several important theories about health-related behaviors, including the health belief model, the theory of reasoned action (TRA) or theory of planned behavior, the social cognitive model, and the KABP (Knowledge-Attitude-Belief-Practice) model (14, 15). The health belief model suggests that people's beliefs about health problems, perceived benefits of action and barriers to action and self-efficacy explain engagement (or lack of engagement) in health-promoting behaviors. The TRA is used to predict how individuals behave based on their pre-existing attitudes and behavioral intentions. The social cognitive and KABP models are social learning and imitation theories. These theories discuss perceived susceptibility of threat of risk behaviors, and benefits, barriers and efficacy in changing risk behaviors. They can be used for exploring individual motivations (such as attitudes, belief and perception) and identifying ways to target individuals based on their motivations or perceived barriers to emergency preparedness.

**Figure 1:**
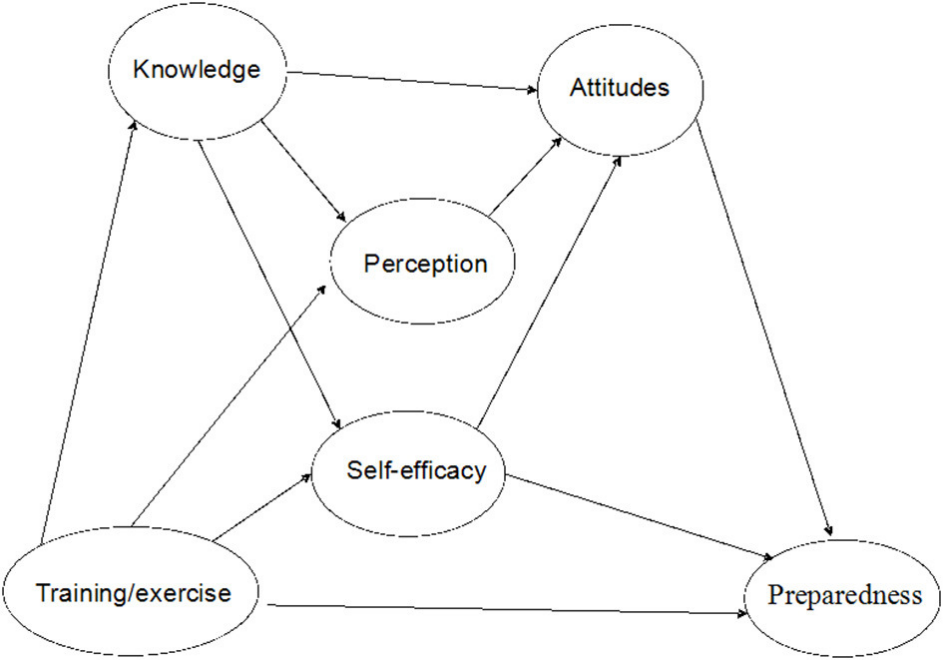
Theoretical model for emergency preparedness behaviors.

In line with the above theories, we developed a theoretical model (Figure 1) with the following hypotheses:

1. Individual emergency preparedness behaviors are associated with individual attitudes, self-efficacy (the extent or strength of one's belief in one's own ability to complete tasks and reach goals), and training/exercise for emergency response (practical skills for completing tasks).
2. Individual attitudes toward emergency preparedness are associated with knowledge, risk perception, and self-efficacy.
3. Individual perception of risks is associated with level of knowledge, which can change through training and drill exercise.
4. Self-efficacy is associated with level of knowledge and can be improved through training and drill exercise.

## Methods

This study tested goodness-of-fit of the above theoretical model for emergency preparedness behaviors using structural equation modeling.

### Participants

The study was conducted in three provinces of China: Guangdong, Heilongjiang and Sichuan. They were chosen purposively to ensure diversities in geographical location, climate and economic development.

A stratified cluster sampling strategy was adopted to select study participants. In each province, the capital city and a medium-sized city were selected. Then an urban district and a rural county were selected randomly in each city. This was followed by a random sampling of two residential communities/villages within each selected district/county. Finally, the vast majority of households in the selected communities/villages were approached and invited to participate in the study.

One family member aged over 16 years old from each participating household responded to the questionnaire interview.

### Data Collection

Data were collected from April to October 2017. Postgraduate students from the School of Public Health of Harbin Medical University were recruited and trained to conduct face-to-face questionnaire interviews. Each questionnaire interview took on average 20 min to complete. This study was approved by the institutional review board of Harbin Medical University (Number: HMUIRB20150016).

In total, 2,625 questionnaires were completed including 850 in Guangdong, 900 in Heilongjiang and 875 in Sichuan. The questionnaires containing missing data were excluded for data analyses. This resulted in a final sample size of 2,506, representing 95.5% of the returned questionnaires.

### Instrument

The questionnaire was first developed by Enders (10), which had been tested in a Harbin population prior to the survey with acceptable reliability and validity (16). The Cronbach's α of the questionnaire reached 0.818 in this study.

### Measures

#### Individual Emergency Preparedness Behaviors

Two question items were developed to measure preparedness. One measured common emergency supplies (yes or no) at home. The US Department of Homeland Security recommends five essential items (food and bottled water for 3-day supply, first aid kit, battery-powered radio, flashlight, and extra batteries) as ideal preparedness (scored 1 or 0 otherwise) (17). Another item described the current status of emergency preparedness. Respondents were classified into one of the five categories:“having no plan to take any action,” “planning to start preparation in the next 1-6 months,” “having started to think about emergency preparedness action,” “having partly completed emergency preparedness action,” “having been fully prepared for all kinds of emergency actions over the past 6 months.” The score ranged from 1 to 5 (highly prepared).

#### Knowledge

Knowledge levels related to four types of emergency events (natural disaster, accident, public health and terrorist attack) were assessed, each containing four question items. For example, the respondents were asked “Which place is not safe in a house during an earthquake?” A correct answer was given a score of 1; whereas an incorrect answer or “don't know” scored 0. The item scores for each type of emergency event were added, generating a summed score ranging from 0 to 4.

#### Attitudes

Three question items measure individual attitudes toward emergency events: individual interests in accessing emergency information, active engagement in preparedness conversations, and attention to emergency alerts. The respondents were asked to rate their attitudes from 1 to 5, with a higher score indicating a higher level of interest/attention.

#### Perception

The respondents were asked to rate the likelihood of occurrence of the four types of emergency events from 1 to 5,with a higher score indicating a higher level of agreement with the likelihood of the emergency event.

#### Self-Efficacy

Self-efficacy has been suggested to be critical for the successful maintenance of changing behaviors (18). Two question items were included in this study to measure self-efficacy in relation to emergency rescues (e.g., confidence in emergency actions) and emotional response (e.g., maintaining calm in emergency events). The respondents were asked to rate self-efficacy from 1 to 5, with a higher score indicating higher self-efficacy.

#### Training and Exercise

The respondents were asked whether they had attended any training/education programs (yes or no) and/or participated in drill exercises (yes or no).

### Data Analysis

We tested the hypothesized theoretical model using structural equation modeling (SEM). SEM allows us to examine a set of relationships among one or more variables, either continuous or discrete. The variables can be a latent construct (a variable that is not directly observed but is rather inferred from other variables) or an observed variable (directly measured). It can display all potential pathways, with each representing a hypothesized relationship with the direction of effect identified as either positive or negative.

The goodness of fit of the models was assessed using the following indicators: Comparative Fit Index (CFI), Adjusted Goodness of Fit (AGFI), Normed Fit Index (NFI), Tucker Lewis Index (TLI), and Root Mean Square Error of Approximation (RMSEA). While the χ^2^ value is likely to yield a model rejection in large samples, other traditional model fit indicators can provide information independent of the sample size.

**Figure 2:**
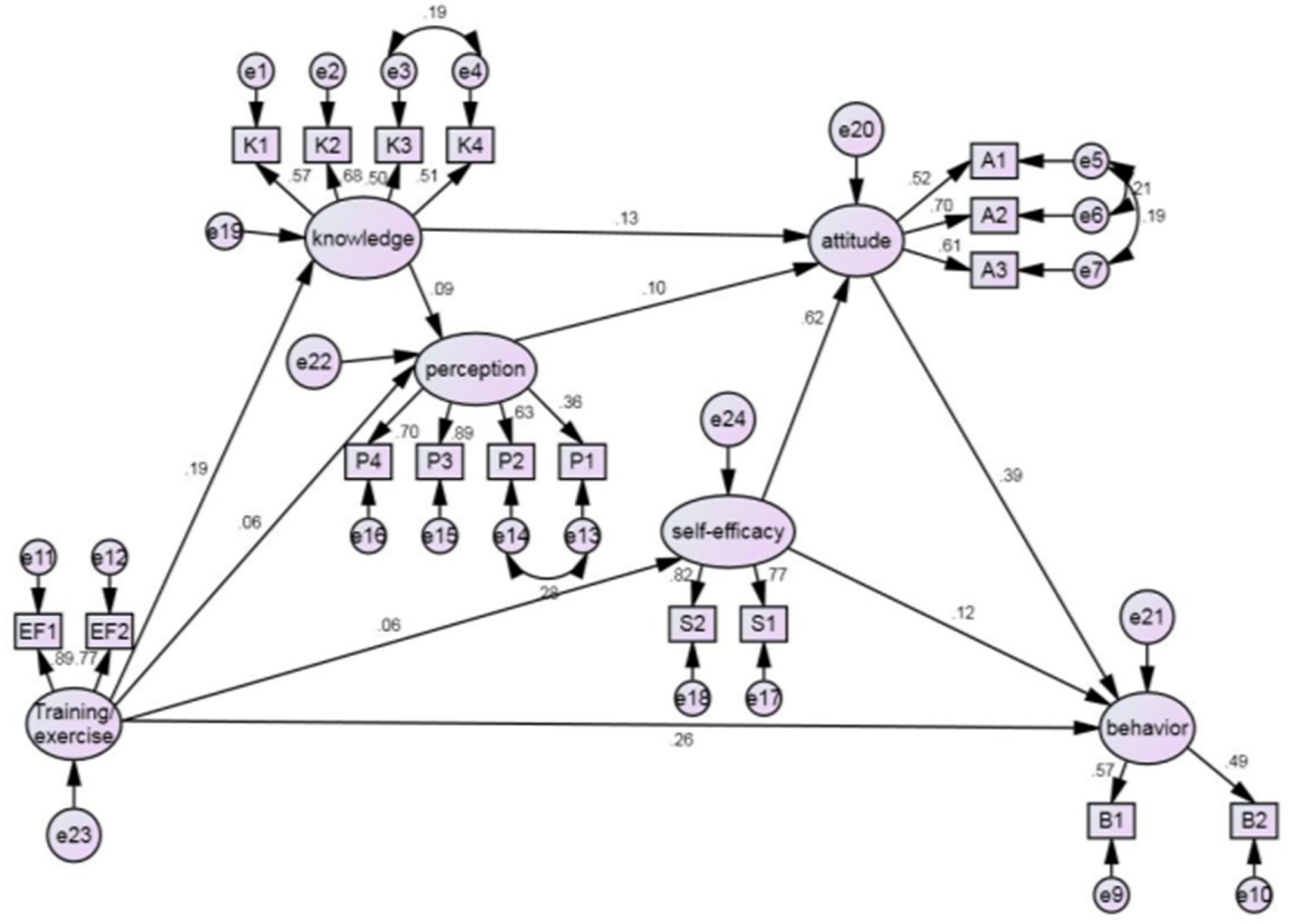
The modified emergency preparedness behavior model.

The original hypothesized model showed statistical significance for the path between self-efficacy and behaviors (*p* < 0.05). Further testing indicated that the link between knowledge and self-efficacy was statistically insignificant (*p* = 0.834, *p* > 0.05). The path between self-efficacy and knowledge was subsequently dropped in the final model (Figure 2).

We presented standardized factor loadings (β) in the final model (Figure 2), which can be interpreted as correlation coefficients. The factor loadings exhibit the strength of correlations between latent constructs and between the observed variables and their corresponding latent constructs. We also calculated direct effects, indirect effects and total effects for each of the latent constructs on emergency preparedness behaviors.

All of the statistical analyses were performed using the SPSS statistical software package (version 19) and the AMOS.

## Results

### Characteristics of Respondents

More than 66% of respondents were in the age range between 31 and 50 years. Women accounted for slightly over half (53%) of the respondents. The majority (83.8%) of respondents were married at the time of the interviews. About 16% of respondents had obtained a university qualification (Table 1).

**Table 1:** Demographic characteristics of respondents.

**Characteristics**	**Freq. (%)**
**Gender**	
Male	1,177 (47.0)
Female	1,329 (53.0)
**Level of education**	
Middle school or below	1,136 (45.3)
High school	369 (14.7)
College	595 (23.7)
Bachelor degree or above	406 (16.2)
**Marital status**	
Married	2,099 (83.8)
Others	407 (16.2)
**Age (years)**	
≤ 30	584 (23.3)
31–50	1,664 (66.4)
≥51	258 (10.3)
**Residency**	
Urban	1,306 (52.1)
Rural	1,200 (47.9)
**Average household monthly income (CNY)**	
0–2,000	750 (29.9)
2,001–5,000	982 (39.2)
≥5,001	774 (30.9)

*N = 2,506; Freq., frequency*.

### Individual Emergency Preparedness Behaviors

Only 6.7% of respondents reported having a 3-day supply of five essential items. About 5.1% of households were fully prepared for emergency events, compared with 25.6% having no plan at all, 23% being partly prepared, and the rest intending or considering to take action (20.5% thinking about actions; 25.8% planning to start later).

### Latent Constructs and Observed Variables in the Model

Overall, the knowledge scores of respondents ranged from 2.71 to 3.67 (out of a total of four), the mean score for earthquake was 2.71 ± 1.12, fire was 3.67 ± 0.70, infectious diseases was 2.86 ± 0.92 and terrorism was 3.16 ± 0.99.

The perceived risk of four types emergency events was low, with 69.2% of respondents chose terrorist attack as the lowest possibility among four types of emergency events (Table 2).

**Table 2:** Perception, attitudes and self-efficacy of respondents toward emergency preparedness.

**Dimensions**	**Very low *N* (% of 2,506)**	**low *N* (% of 2,506)**	**Average *N* (% of 2,506)**	**High *N* (% of 2,506)**	**Very high *N* (% of 2,506)**
**Perception**					
Likelihood of natural disaster	556 (22.2)	994 (39.7)	532 (21.2)	241 (9.6)	183 (7.3)
Likelihood of accident	363 (14.5)	896 (35.8)	829 (33.0)	343 (13.7)	75 (3.0)
Likelihood of public health emergencies	432 (17.2)	1,029 (41.1)	773 (30.8)	205 (8.2)	67 (2.7)
Likelihood of terrorist attack	583 (23.3)	1,151 (45.9)	567 (22.6)	150 (6.0)	55 (2.2)
**Attitudes**					
Interest in emergency information	131 (5.2)	210 (8.4)	932 (37.2)	766 (30.6)	467 (18.6)
Emergency preparedness topics	113 (4.5)	288 (11.5)	972 (38.8)	769 (30.7)	364 (14.5)
Attention to emergency alerts	92 (3.7)	108 (4.3)	559 (22.2)	846 (33.8)	901 (36.0)
**Self-efficacy**					
Self-efficacy of emergency rescue	107 (4.3)	311 (12.4)	1,157 (46.2)	645 (25.7)	286 (11.4)
Emotional response	72 (2.9)	304 (12.1)	1,082 (43.2)	711 (28.4)	337 (13.4)

Most respondents show the great interest toward emergency preparedness activities, and would like to share the Emergency preparedness topics with their family members. About 69.8% of respondents shows high attention to emergency alerts from local government announcement (Table 2).

About 46.2% of respondents hold their Self-efficacy of emergency rescue is moderate level, and 41.8% respondents thought they can keep calm when they suffer emergency events (Table 2).

About 25% of respondents attended emergency training programs. But only a small percentage (17%) participated in drill exercises.

The modified model for emergency preparedness behaviors (Figure 2) demonstrated a good fit with the data, CFI = 0.973, GFI = 0.983, and RMSEA = 0.031.

The 10 pathways illustrated in the modified model (Figure 2) were statistically significant, all having a positive factor loading. Attitudes, training/exercise and self-efficacy were associated with behaviors. Attitudes had a stronger association with emergency preparedness behaviors (β = 0.385, *p* < 0.001) than training/exercise (β = 0.263, *p* < 0.001). The hypothesized association between self-efficacy and emergency preparedness behaviors was statistically significant (β = 0.119, *p* = 0.018 <0.05). Hypothesis 1 was supported.

Attitudes were associated with self-efficacy (β = 0.618, *p* < 0.001), knowledge (β = 0.131, *p* < 0.001) and risk perception (β = 0.097, *p* < 0.001). Hypothesis 2 was therefore supported. The strong association between self-efficacy and attitudes enabled self-efficacy to impose a strong total effect (0.357) on emergency preparedness behaviors (0.119 direct effects and 0.238 indirect effects) (Table 3).

**Table 3:** Standardized direct, indirect, and total effects of factors on emergency preparedness behaviors.

**Construct**	**Total effects**	**Direct effects**	**Indirect effects**
Attitudes	0.385	0.385	0.000
Self-efficacy	0.357	0.119	0.238
Training/exercise	0.296	0.263	0.033
Knowledge	0.054	0.000	0.054
Perception	0.037	0.000	0.037

Knowledge was associated with training/exercise (β = 0.188, *p* < 0.001).

Risk perception was associated with knowledge (β = 0.090, *p* = 0.001) and training/exercise (β = 0.059, *p* = 0.017). Hypothesis 3 was supported.

Self-efficacy was associated with training/exercise (β = 0.058, *p* = 0.020). But the hypothesized link between self-efficacy and knowledge was statistically insignificant (*p* = 0.834). Hypothesis 4 was not fully supported.

## Discussion

This study revealed that personal emergency preparedness behaviors are associated with knowledge, attitudes, risk perception, self-efficacy, and training/exercise. These findings are consistent with the psychological theories derived from other studies.

We found that attitudes are the strongest predictor of emergency preparedness behaviors. Attitudes may also serve as a mediator for the effects of knowledge, risk perception, and self-efficacy on preparedness behaviors. Previous studies have identified attitudes as a key determinant of emergency preparedness (19–21). According to the Theory of Planned Behavior; intention is the proximal determinant of behaviors, which is shaped by attitudes (22, 23).

The finding about the association between self-efficacy and preparedness behaviors came as a surprise: the direct link between self-efficacy and preparedness is statistically insignificant. Never the less, the indirect effect of self-efficacy on preparedness behaviors is statistically significant and profound. A higher level of self-efficacy is associated with more positive attitudes toward preparedness. The literature claims that people with higher self-efficacy always perform better in action (24). Self-efficacy can be fostered through directive experience alternative experience, verbal persuasion and emotional response (25, 26).

Training and drill exercise has been adopted as a common strategy for improving emergency preparedness. It is believed that training/exercise can raise the awareness of the public and help them improve their knowledge, foster positive attitudes, and develop practical skills in preparing for emergencies (27, 28). It is evident that appropriate training and drill exercise can reduce casualties of disasters (29, 30). However, our study demonstrates that the effects of training/exercise on individual preparedness are predominantly direct effects (0.263). The indirect effects of training/exercise on preparedness are relatively weak (0.033). It is likely that training and exercise improves the individual ability to successfully cope with problem solving in the case of emergencies.

Knowledge has been widely regarded as an indicator for emergency management capability. According to the KABP model, knowledge is one of the critical factors affecting behaviors (31, 32). But we found that the effect of knowledge on preparedness behaviors is quite weak, albeit statistically significant.

We also noted that risk perception has a weak indirect effect (0.037) on preparedness behaviors. Perception interventions are often adopted as a first step to encourage people to up take preparedness activities. Previous studies (33–35) have attempted to establish the association between risk perception and behaviors. Risk perception depends on an individual understanding of the emergency events (36–38). A lack of awareness of risks can jeopardize emergency preparedness (39).

### Limitations

There are certain limitations in this study. First, the questions measuring emergency preparedness were intentionally broad, which may cause unclear or vague responses from some participants, especially those with a low level of education. Second, we measured perceived likelihood of emergency events. However, it may not necessarily reflect perceived threats and consequences of the emergency events. Caution needs to be taken when interpreting the weak link between risk perception and preparedness behaviors. Further studies are warranted, taking into consideration the perceived seriousness of emergency events.

In terms of the sampling approach, our study was limited by the difficulty of implementing face-to-face interview. We identified three provinces after considering the diversities in geographic location and socioeconomic development. Guangdong represents the most developed province in eastern China, most frequently affected by typhoons. Meanwhile, many infectious diseases erupted in Guangdong, such as Severe Acute Respiratory Syndrome in 2003 and dengue fever in 2014. Heilongjiang and Sichuan represent the less developed regions in central and western China, respectively. Sichuan is an earthquake-prone area and experienced the Wenchuan earthquake in 2008. Although these three provinces are relatively representative, it is still necessary to be cautious about whether the conclusion is applicable to the whole China.

If this study had been conducted in 2020–2021, the findings might have been somewhat different. For example, since the outbreak of COVID-19 in 2019, our research team has focused on this public health emergency of international concern and we found some changes in the individual emergency preparedness behaviors.

## Conclusion

Individual emergency preparedness behaviors are associated with attitudes, training/ exercise, self-efficacy, knowledge, and risk perception. Attitudes have the greatest impact on preparedness behaviors, followed by training/exercise and self-efficacy. Knowledge and risk perception are indirectly linked with preparedness behaviors. This suggests that individual emergency preparedness can be improved through changing attitudes and training/exercise plays an important role in enhancing preparedness behaviors both directly and indirectly (through improving self-efficacy, attitudes and knowledge).

## Data Availability Statement

The original contributions generated for this study are included in the article/Supplementary Material, further inquiries can be directed to the corresponding author/s.

## Ethics Statement

The studies involving human participants were reviewed and approved by the institutional review board of Harbin Medical University (Number: HMUIRB20150016). The patients/participants provided their written informed consent to participate in this study.

## Author Contributions

NN, MH, and JQ: study and manuscript conceptualization. NN and CL: contributed to methods. NN, XZ, WeiX, WeilX, BZ, ZC, YY, YH, and QW: contributed to background, methods, results, and discussion. All authors contributed to the article and approved the submitted version.

## Conflict of Interest

The authors declare that the research was conducted in the absence of any commercial or financial relationships that could be construed as a potential conflict of interest.
