# Supplementary material for: Factors Associated With Individual Emergency Preparedness Behaviors: A Cross-Sectional Survey Among the Public in Three Chinese Provinces
Source: Front Public Health. 2021 May 21;9:644421. doi: 10.3389/fpubh.2021.644421 (PMC8175617; doi:10.3389/fpubh.2021.644421)
Supplement: Supplementary file 2 [file Data_Sheet_1.docx]

Questionnaire on the Status of Public Emergency Preparedness

Dear Friends:

In order to minimize the losses caused by emergencies, governments have attached great importance to the emergency preparation and capacity building of the public in addition to strengthening the team building of professionals in view of the increasingly frequent emergencies. This questionnaire survey aimed to understand the current situation of public emergency preparedness so as to improve the public emergency ability and protect the public's life, health and safety. We are very pleased to invite you to participate in this survey. We guarantee that all your information will be kept strictly confidential and will be used for research only. Thank you for your understanding and support!

**Research Team of Harbin Medical University**

| Instruction  1. Please fill in the answer in the corresponding position and do not miss any questions.  2. Please fill in with your real thoughts, opinions, knowledge, etc  3. Key terms:  **Emergencies:** refer to those that occur suddenly and cause or may cause major casualties, property losses and ecological environment disruption and serious social hazards, emergency events endangering public safety.  Emergencies are divided into four categories: natural disasters, accident disasters, public health, and social safety.  **Emergency preparedness:** refers to the general term of various measures taken in advance to effectively respond to emergencies, including the formulation of emergency planning, learning emergency knowledge and skills, storing emergency materials, participating in education, training and drills, etc., To establish, maintain and improve the public's ability to respond to various emergencies. |
| --- |

**Investigator:**

**Date:**

**Your residence:**① urban ② rural

**1. Gender:** ① male ② female

**2. Age (years):**

**3. Ethnicity:** ① Han ② others

**4. Marital status:** ① unmarried ② married ③ divorced ④ widowed ⑤ others

**5. Education level:**

① Middle school or below ② High school ③ College ④ Bachelor degree or above

**6.Average household monthly income(CNY)** ①0-2000 ②2001-5000 ③≥5001

**7. Have you participated in relevant emergency knowledge training?** ① Yes ② No

**8. Are there common emergency supplies in your home?** ① Yes ② No

□ Gas mask □ Fire extinguisher □ Escape rope □ Outdoor tent □ First aid kit □ Flashlight □ Radio □ Standby battery □ Whistle □ Dust mask □ Warm clothing □ food and bottled water for three-day supply□ Important certificate data and copies

9. Which one describes your current status of emergency preparedness?

①having no plan to take any action

②planning to start preparation in the next 1-6 months

③having started to think about emergency preparedness action

④having partly completed emergency preparedness action

⑤having been fully prepared for all kinds of emergency actions over the past six months

10. The following items are knowledge related to four types of emergency events (natural disaster, accident, public health and terrorist attack). Please write “√”for the correct item, and “×”for the incorrect statement.

| Knowledge Items | answer |
| --- | --- |
| Outdoors people should squat down in an open place when an earthquake occurs |  |
| A large amount of food and water should be fed immediately after rescuing the buried person in the earthquake |  |
| It is necessary to adhere to call for help loudly for a long time if buried during an earthquake |  |
| Evacuate by elevator if a tall building fires |  |
| Cover the mouth and nose with a wet towel to leave if the room is filled with thick smoke during the fire |  |
| Call for help near a window or balcony when there is no way to escape |  |
| Escape as low as possible, preferably squatting along the corner |  |
| The early symptoms of human infection with H7N9 influenza are fever, cough, and little sputum |  |
| Avian feces are the main channel for the transmission of human infection with avian influenza |  |
| Vaccination when necessary |  |
| People with suspicious infectious disease symptoms should accept disease management, investigation, treatment |  |
| Escape on the monitored main road as soon as possible and do not enter the alley to evade |  |
| Being hijacked and shouting for help, and fighting with terrorists |  |
| When hijacked, do not look at, talk, lie on the ground, and move slowly. |  |
| Playing dead is an effective way when resistance is ineffective |  |

**11. The following statements are possible descriptions about you and your community. Please circle one response for each statement.**

**Response options: 1 Strongly Disagree 2 disagree 3 Neither Disagree Nor Agree 4 Agree 5 Strongly Agree**

| 1 | I am very interested in accessing information related to various emergencies | 1 | 2 | 3 | 4 | 5 |
| --- | --- | --- | --- | --- | --- | --- |
| 2 | I am happy to discuss various emergency preparedness topics with my family members/friends | 1 | 2 | 3 | 4 | 5 |
| 3 | I attach importance to the official emergent warning | 1 | 2 | 3 | 4 | 5 |
| 4 | There is a high likelihood of natural disasters (earthquakes, typhoons) occurrence in the place where I live | 1 | 2 | 3 | 4 | 5 |
| 5 | There is a high likelihood of any accident disaster (fire, car accident) occurrence in the place where I live | 1 | 2 | 3 | 4 | 5 |
| 6 | There is a high likelihood of any public health event such as an infectious disease occurrence in the place where I live | 1 | 2 | 3 | 4 | 5 |
| 7 | There is a high likelihood of any violence and terror occurrence in the place where I live | 1 | 2 | 3 | 4 | 5 |
| 8 | I'm so confident to respond to any emergencies effectively | 1 | 2 | 3 | 4 | 5 |
| 9 | I always stay calm when I encounter any emergencies | 1 | 2 | 3 | 4 | 5 |

* Thank you for your participation! *
